# Iron Oxide Mesoporous Magnetic Nanostructures with High Surface Area for Enhanced and Selective Drug Delivery to Metastatic Cancer Cells

**DOI:** 10.3390/pharmaceutics13040553

**Published:** 2021-04-14

**Authors:** Kheireddine El-Boubbou, Rizwan Ali, Sulaiman Al-Humaid, Alshaimaa Alhallaj, O. M. Lemine, Mohamed Boudjelal, Abdulmohsen AlKushi

**Affiliations:** 1 Department of Basic Sciences, College of Science & Health Professions (COSHP), King Saud bin Abdulaziz University for Health Sciences (KSAU-HS), King Abdulaziz Medical City, National Guard Health Affairs, Riyadh 11481, Saudi Arabia; aliri@ngha.med.sa (R.A.); alhumaid538@ksau-hs.edu.sa (S.A.-H.); KushiA@NGHA.MED.SA (A.A.); 2 King Abdullah International Medical Research Center (KAIMRC), King Abdulaziz Medical City, National Guard Health Affairs, Riyadh 11426, Saudi Arabia; alhallajal@NGHA.MED.SA (A.A.); boudjelalmo@ngha.med.sa (M.B.); 3 Department of Physics, College of Sciences, Imam Mohammad Ibn Saud Islamic University (IMISU), Riyadh 11623, Saudi Arabia; leminej@yahoo.com

**Keywords:** magnetic nanoparticles, mesoporous, iron oxide, drug delivery, anticancer drugs, chemotherapy

## Abstract

This work reports the fabrication of iron oxide mesoporous magnetic nanostructures (IO-MMNs) via the nano-replication method using acid-prepared mesoporous spheres (APMS) as the rigid silica host and iron (III) nitrate as the iron precursor. The obtained nanosized mesostructures were fully characterized by SEM, TEM, DLS, FTIR, XRD, VSM, and nitrogen physisorption. IO-MMNs exhibited relatively high surface areas and large pore volumes (*S_BET_* = 70–120 m^2^/g and *V_pore_* = 0.25–0.45 cm^3^/g), small sizes (~300 nm), good crystallinity and magnetization, and excellent biocompatibility. With their intrinsic porosities, high drug loading efficiencies (up to 70%) were achieved and the drug release rates were found to be pH-dependent. Cytotoxicity, confocal microscopy, and flow cytometry experiments against different types of cancerous cells indicated that Dox-loaded IO-MMNs reduced the viability of metastatic MCF-7 and KAIMRC-1 breast as well as HT-29 colon cancer cells, with the least uptake and toxicity towards normal primary cells (up to 4-fold enhancement). These results strongly suggest the potential use of IO-MMNs as promising agents for enhanced and effective drug delivery in cancer theranostics.

## 1. Introduction

Mesoporous magnetic materials have been thoroughly investigated in a wide spectrum of industrial and research areas ranging from catalytic, photonic, and electronic devices and batteries to biological, biomedical, and drug delivery applications [1,2]. It is well recognized that mesoporous materials exhibit exceptional unique properties that emerge as a consequence of their small sizes, high surface areas, large pore volumes, and tunable pore structures [3]. The functionality of such materials can be significantly improved when their sizes are confined to the nanoscale and their morphologies are controlled to have a high surface area. In addition, since mesoporous magnetic materials are both magnetic and porous, their use in medicine as drug carriers, imaging agents, and hyperthermia vehicles is highly appealing [4,5]. In fact, controlled drug delivery systems are incessantly explored due to the promising potential to enhance the therapeutic efficacy of chemotherapeutic drugs and reduce the systemic conventional side effects in cancer treatments. Of the various drug delivery systems [6,7], mesoporous magnetic nanomaterials are especially attractive, because they can entrap large amounts of drug molecules in their pores, and subsequently release them inside the cells. In addition, their minimal toxicity, stability, and biocompatibility due to the iron oxide composition make such mesostructures particularly unique for clinical applications. Magneto-based particles of similar composition have been enormously used as magnetic imaging probes, hyperthermia agents, and magnetic-guided drug delivery carriers for cancer theranostics [8]. Nevertheless, despite such distinctive potentials, fabrication of nanosized mesostructures of iron oxides containing large surface areas and pore volumes remain extremely challenging.

Many synthetic routes have been suggested to produce nano-scaled mesostructures of iron oxides. In fact, the majority of research has focused on preparation of iron oxide magnetic nanoparticles (IO-MNPs) coated with mesoporous silica (core/shell) or embedded in silica matrix [9,10,11,12]. The challenges and difficulties encountered in the preparation of mesoporous magnetic metal oxides are well documented [13], with limited improvements in recent years [14]. The synthetic methods for their formation are largely classified into two major routes: Soft and hard templated methods [15,16,17,18]. The soft template process employs the self-assembly induced by amphiphilic block copolymers or surfactants which involves complex sol-gel chemistries and typically leads to materials with low surface areas and pore volumes [19]. On the other hand, in the hard template approach, guest materials replicate the inverse structures of the inorganic host, producing a mesostructured replica [20,21,22,23]. This is preferred over the soft techniques due to the abrupt gelation characteristics, difficulties in the direct derivation into homogeneous crystalline phase, and frequent collapse of mesostructures during the synthesis [24]. In particular, mesoporous silica, with ordered or disordered channels, which possess uniform pores, remarkably high surface areas, and high silanol densities, act as an ideal host for the fabrication of nanostructured mesoporous metal oxides [25,26,27,28,29]. For instance, iron (III) salts were used as precursors that can either co-precipitate with the silica source or infiltrate into porous silica, yielding IONPs embedded inside the silica matrix after thermal treatments [30,31]. Moreover, synthesis of ordered and disordered mesoporous Fe_3_O_4_ and Fe_2_O_3_ with crystalline walls have been elegantly reported using mesostructured 3D bicontinuous KIT-6 and 2D SBA-15 as the silica templates [32,33,34,35]. However, according to our knowledge, very few reports have prepared mesoporous iron oxide nanoparticles for therapeutic drug delivery applications [36,37,38]. Recently, we synthesized IO-magnetic mesoporous microparticles co-loaded with the hydrophilic chemotherapeutic drug (daunorubicin) and the hydrophobic hormonal drug (tamoxifen), and showed enhanced synergistic anticancer chemo/hormonal therapy in both cancerous cells and patient tumor biopsies [39].

Herein, we report the preparation of iron oxide mesoporous magnetic nanostructures (IO-MMNs) via nano-replicated impregnation-etching process using acid-prepared mesoporous spheres (APMS) as the silica host and iron (III) nitrate as the iron precursor. The fabricated IO-MMNs exhibited uniform spherical morphologies, high surface areas, large pore volumes, and controllable pore structures, especially beneficial for enhanced therapeutic drug delivery. In vitro release studies, cytotoxicities, intracellular uptake, and abilities to kill different types of cancerous cells were systematically evaluated. The anticancer drug-loaded IO-MMNs established in this work can potentially open new opportunities for in vivo selective and directed cancer theranostics.

## 2. Materials and Methods

### 2.1. Materials

Unless otherwise indicated, all chemicals and solvents were obtained from commercial suppliers and used as received without further purification. Iron (III) nitrate nonahydrate (Fe(NO_3_)_3_·9H_2_O), zinc metal powder, cetyltrimethylammonium bromide (CTAB), tetraethyl orthosilicate (TEOS), sodium fluoride (NaF), conc. HCl, dimethyl sulfoxide (DMSO), ammonium hydroxide (NH_4_OH), and the anticancer drug Doxorubicin (Dox) were all purchased from UFC Biotechnology (Riyadh, Saudi Arabia). Dulbecco’s phosphate buffered saline (DPBS), phosphate buffered saline (PBS), advanced Dulbecco’s modified eagle medium (DMEM), phenol red-free DMEM, fetal bovine serum (FBS), Hoechst 33,342 stain, l-glutamine, and penicillin-streptomycin (Pen-Strep) were all purchased from Invitrogen or UFC Biotechnology. MTT (thiazolyl blue tetrazolium bromide) was purchased from Bioworld (Visalia, CA, USA). All cell lines were purchased from the American Type Culture Collection (ATCC) and grown in RPMI 1640 medium supplemented with 10% FBS and 1% penicillin/streptomycin. Human cancerous cells used in this study are: MCF-7 (Michigan Cancer Foundation-7 metastatic breast cancer cell line isolated from a 69-year-old Caucasian woman), KAIMRC-1 (naturally transformed breast cancer cell line from an Arab woman of age 62 suffering from stage IIB breast cancer isolated from one of the primary breast tumor tissue), HT-29 (human colon adenocarcinoma cell line isolated from the primary tumor of a 44-year-old Caucasian female), and primary normal fibroblast-like cells used as controls.

### 2.2. Characterization

SEM images were processed using an FEI NanoSEM 450 scanning electron microscope at 15 kV. TEM images were collected on a JEOL-JEM 1400 operating at 120 kV using Gatan camera with TEM Center Ver.1.4.3222 (TEM Operating & Acquisition System). FTIR spectra (400–4000 cm^−1^) were recorded as KBr pellets using Shimadzu IRAffinity-1. DLS and zeta potential measurements were assessed on Malvern Zetasizer Nano ZS instrument. X-ray powder diffraction (XRD) measurements were performed using Bruker D8 Discover diffractometer (θ–2θ) equipped with Cu-Kα radiation (λ = 1.5406 Å). Magnetic measurements were measured at room temperature using LakeShore 7404 model vibrating sample magnetometer (VSM) having a 1.8 Tesla magnet. Calcinations were carried out under flowing air atmosphere. The following heating profile was used for calcinations: 2 °C/min ramp to 550 °C with 4 h hold at 550 °C. Flow cytometry measurements were assessed using BD FACSCanto^TM^ II Flow Cytometer. Confocal images were visualized using inverted Zeiss LSM 780 multiphoton laser scanning confocal microscope equipped with 20× and 40× (oil immersion) objectives and axiocam cameras.

### 2.3. Preparation of IO-MMNs

APMS silica template was first prepared following our previous work [40,41] using CTAB (10 g), H_2_O (200 mL), conc. HCl (20 mL), TEOS (20 mL), and ethanol (70 mL). NaF (0.5 M in water, 20 mL) was then added and stirring was continued until turbidity was reached. The mixture was then quickly transferred to a Teflon bottle and heated at 100 °C for 120 min. The resulting APMS precipitate was filtered, washed with water and ethanol, and dried under vacuum. Calcination at 550 °C using 2 °C/min ramp under air afforded calcined APMS ready for iron impregnation, and 200 mg of APMS in 10 mL water was sonicated, followed by the addition of 1 g of Fe(NO_3_)_3_·9H_2_O aqueous solution (5 mL). Trace amounts of zinc metal were then added, and the reaction mixture was vigorously stirred for 24 h at room temperature. A change in color was observed and the resulting material was decanted to separate the zinc, purified by repeated centrifugation/washing steps with water and ethanol (ethanol being the final wash), and finally dried under vacuum. This process was then repeated using 0.85 g of Fe(NO_3_)_3_·9H_2_O solution to ensure complete iron impregnation. The second iron impregnation is crucial, as it allows the complete filling of the mesopores. The obtained product was then heated slowly to 350 °C at 2 °C/min and then left at this temperature for 3 h. Finally, the sample was treated twice with hot 5 M NaOH solution to remove the silica template, purified by centrifugation, washed with water and ethanol, and then dried to afford IO-MMNs.

### 2.4. Drug Loading and Release

One milliliter of aqueous dispersion of IO-MMNs (1.0 mg/mL) and aqueous Dox solution (200 μg/mL) were gently shaken on a rotary shaker for 36 h. Dox@IO-MMNs were then isolated via centrifugation, washed repeatedly with water until no drug was detected in the supernatant, and finally re-dispersed in water. Loading efficiencies were determined by UV-vis spectroscopy. The absorbance of the residual drug in the supernatant was measured (λ_max_ = 490 nm for Dox) and the percentage of drug loading (*w/w*%) was then quantified as in our previous reports [42,43]. For drug release, 5 mg of Dox@IO-MMNs were suspended in 1 mL PBS buffer at two different pH values (pH = 7.4 and pH = 4.5) and gently rotated at 37 °C. At specific time points, supernatants were taken from each tube, measured by UV-vis, and returned to their respective tubes after measurements. The percent drug release was calculated as per our earlier work [42,43].

### 2.5. Cell Viability Assay

Cell viability of the different cancer cells exposed to IO-MMNs was determined using MTT assay. The cell lines were seeded in a 96-well plate at a density of 5 × 10^4^ cells/well and incubated in 95%/5% humidified air/CO_2_ at 37 °C. After 24 h, the media was removed and fresh phenol red-free DMEM containing 0.5% FBS was added to the cells. Cells were then treated with equivalent concentrations of IO-MMNs, Dox@IO-MMNs, or free Dox (15, 12.5, 10, 7.5, 5, and 1.5 μL from the prepared tubes) in 200 μL supplemented DMEM. After 24 h of incubation, the media was removed, and the cells were washed with PBS. Cell viability was then determined using the MTT viability assay following the manufacturer’s protocol. Experiments were conducted in triplicates and mean averages with standard deviations were plotted.

### 2.6. Live Confocal Imaging

Cells were incubated in an 8-well dish for 24 h prior to IO-MMNs exposure. After removing the supernatant, cells were then exposed to Dox@IO-MMNs, free Dox, or free IO-MMNs at equivalent concentrations incubated for different periods of time (6 and 24 h). The cells were then stained with Hoechst 33,342 and/or Calcein (when needed) and then allowed to settle down for 20 min before live microscopic visualization (no fixation).

### 2.7. Fluorescence-Activated Cell Sorting (FACS)

First, FITC@IO-MMMs were prepared following literature procedure. Briefly, 22 μL of FITC was reacted with 22 μL of APTES in 1 mL ethanol for 2 h in the dark. Then, 50 μL of the above solution was added to 1 mL of IO-MMMs (1 mg/mL) followed by the addition of 50 μL of NH_4_OH. The reaction was shaken for 48 h, centrifuged, and then washed several times with ethanol and water until no FITC was detected in the supernatant. Cell lines (1 × 10^5^ cells per well) were seeded in 6-well culture dishes, incubated for 24 h, and then incubated with FITC@IO-MMMs (final concentration 35 μg/mL) for different time periods. Cells were collected, pelleted, washed with PBS twice, and then transferred to FACS tubes for analysis. Flow cytometry measurements were then assessed in triplicates.

## 3. Results and Discussion

### 3.1. Preparation and Characterization of IO-MMNs

**Figure 1:**
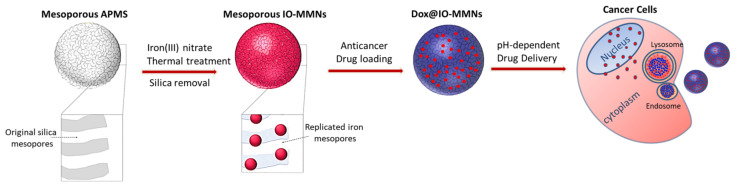
Schematic illustration for the preparation of iron oxide mesoporous magnetic nanostructures (IO-MMNs) from an acid-prepared mesoporous sphere (APMS) silica template and their subsequent loading with anticancer drugs for pH-dependent drug delivery.

The detailed synthetic procedure and therapeutic application of IO-MMN nanoplatform is illustrated in Figure 1. The construction of IO-MMNs is based on the nano-replication wet-etching method [39], using APMS as the silica host and iron(III) nitrate as the iron precursor. Briefly, APMS (~2 μm and 5 nm pore diameters) was first synthesized [41], followed by in situ reduction of iron(III) salt precursors (i.e., aqueous solution of Fe(NO_3_)_3_) during impregnation process, subsequent thermal heating, and finally etching the silica with strong base to afford IO-MMNs (Figure 1). Iron precursors fill the continuous 3D mesopore system, which are subsequently replicated to spherical iron oxide nanoclusters within the silica template during the drying and heating treatments. Interestingly, it was remarked that when iron(III) nitrate was used instead of iron(III) chloride as the iron source, smaller sized IO-MMNs compared to IO-MMMs were obtained. This is similar to earlier observation where Fe(III) nitrate used as the iron precursor produced smaller sized iron oxide (~20 nm) compared to iron oxide nanoparticles (sizes ~120 nm) obtained from the Fe(III) chloride precursor [44].

**Figure 2:**
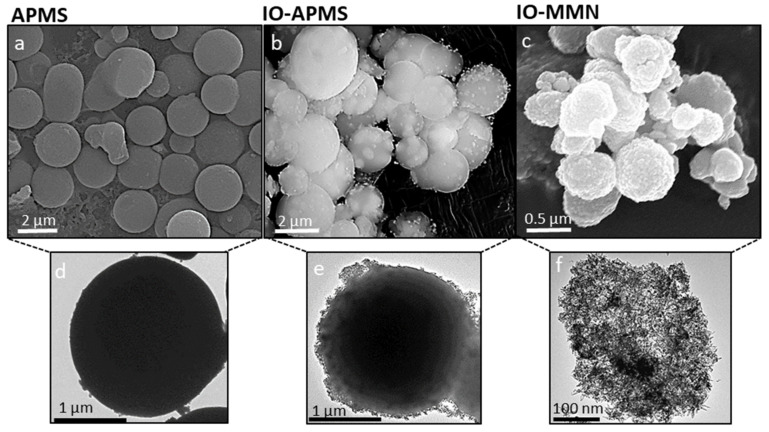
Scanning and transmission electron microscopy images. SEM images of (**a**) APMS silica template, (**b**) iron impregnated APMS (IO-APMS), and (**c**) IO-MMN replicated structures. Respective TEM images of (**d**) APMS, (**e**) IO-APMS, and (**f**) IO-MMN. The electron microscopy images remarkably show the successful formation of iron oxide mesostructures.

**Figure 3:**
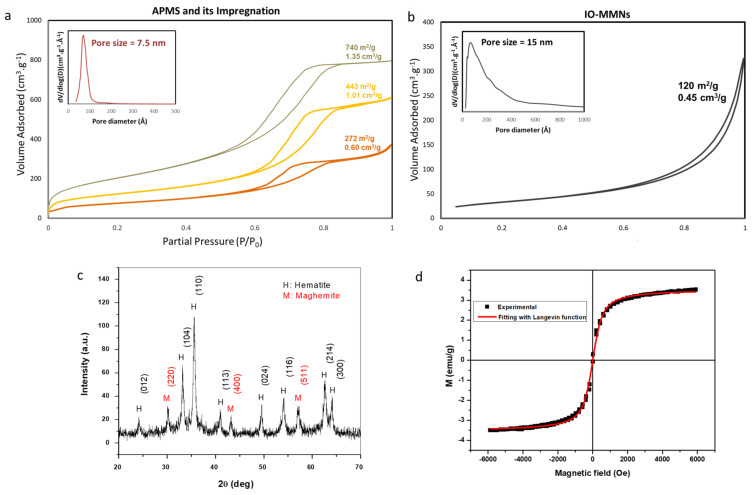
Nitrogen physisorption isotherms for (**a**) APMS and its sequential loading with iron; (**b**) final IO-MMNs after etching and removal of the silica template (inset: Corresponding pore size distribution plots). (**c**) Wide-angle XRD pattern of IO-MMN. (**d**) Field-dependent magnetization hysteresis loops of IO-MMN at room temperature fitted with Langevin function. The results depict the successful formation of IO-MMNs with high surface area and controllable pore volumes, excellent crystallinity, and superparamagnetic nature.

The morphology and size for IO-MMNs were characterized by scanning electron microscopy (SEM) and transmission electron microscopy (TEM). Figure 2a–c shows SEM images of APMS, IO-impregnated APMS, and the final replicated IO-MMNs at different magnifications. A typical averaged sized APMS (~1.5–2 μm) with a spherical-like morphology was obtained. IO-APMS intermediate indicated iron oxide (as dots) infused on the APMS spheres. IO-MMNs, on the other hand, appeared to have bumpy-like structures with sizes about 250–350 nm. TEM images were then conducted to better elucidate the structures at different steps. From the TEM images (Figure 2d–f), it is evident that the original mesoporous silica system is replicated and that the obtained constructs possess mesopores of nanoclustered iron oxide. While the APMS surface appeared unblemished, IO-APMS intermediate showed iron oxide clustering within the pores and on the surface of APMS. Appealingly, IO-MMNs strikingly display the mesostructured morphology, indicating the disordered interconnected channels present in silica template before etching. Next, the mesoporous nature of IO-MMNs was examined by nitrogen physisorption isotherms. As seen in Figure 3a, sequential iron impregnation indicated a progressive decrease in the Brunauer-Emmett-Teller surface area (*S_BET_*) and pore volume (*V_pore_*) compared to the original APMS sample having *S_BET_* = 740 m^2^/g and *V_pore_* = 1.35 cm^3^/g. These results clearly indicate that the iron oxide precursors are incorporated and impregnated inside the inner pore surfaces of the silica. Nitrogen physisorption isotherms showed that the final replicated IO-MMNs exhibit quite high *S_BET_* of 120 m^2^/g and *V_pore_* of 0.45 cm^3^/g (Figure 3b).

Type IV isotherm pattern is clearly observed for IO-MMNs, consistent with reported porosities for mesoporous iron oxide materials. The inset of Figure 3a,b displays Barrett, Joyner, and Halenda (BJH) plots indicating pore size distributions estimated from desorption isotherms of each sample. A BJH plot for IO-MMNs is observed to have a major average pore size diameter of ~15 nm that is broader than the samples before etching (*d* ~7.5 nm). The decrease of pore volume accompanied with an increase in pore diameter indicates that the IONPs were generated within the internal pores with formation of interparticle spaces.

It is worth pinpointing that this experimental method was repeated several times using different APMS samples to obtain IO-MMNs having *S_BET_* = 70–120 m^2^/g, *V_pore_* = 0.25–0.45 cm^3^/g, and *d_pore_* = 15–30 nm. According to our knowledge, the IO-MMNs obtained here possess relatively higher surface areas and controllable pore volumes compared to other iron oxide mesostructured nanomaterials reported in the literature [27,34,45,46]. Fourier-transform infrared (FTIR) was then extended to identify the inorganic composition of IO-MMNs. Similar to our earlier observation [39], FTIR spectroscopy for the obtained IO-MMNs confirmed the characteristic absorption stretching bands of iron oxide Fe-O (~535 and 600 cm^−1^) and O-H (~3200–3600 cm^−1^), pinpointing the formation of iron oxide (i.e., Fe_2_O_3_). To better understand which phase of iron oxide we have, X-ray diffraction (XRD) of IO-MMNs were conducted. As can be seen from Figure 3c, the sample is a mixture of hematite (α-Fe_2_O_3_) and maghemite (γ-Fe_2_O_3_) phases. The most dominant crystalline phase is α-Fe_2_O_3_ (JCPDS No. 79-0007), while the presence of (220), (400), and (511) peaks confirm the presence of γ-Fe_2_O_3_ (JCPDS No. 39-1346) [47]. This is probably due to thermal heating conditions conducted under normal air conditions without inert argon. Magnetization-field (M-H) curves for the synthesized IO-MMNs were recorded at room temperature of 300 K (Figure 3d). It can be seen that the magnetization curve had almost zero retentivity and coercivity, indicating its superparamagnetic nature. This is crucial as in the presence of an external magnetic field (H), these particles could be easily magnetized to reach their saturation magnetization (M_S_), but when H was removed, their overall magnetic moment dropped back to zero, making them extremely stable in dispersion states and ready to be used for biomedical applications. In order to confirm the superparamagnetic nature of the obtained IO-MMNs, the experimental magnetization values were fitted with Langevin theory of paramagnetism using the following equation:

$$M\left(H,T\right)=M_{S}\lfloor \coth\left(\frac{m_{\mathrm{NP}}H}{k_{B}T}\right)-\frac{k_{B}T}{m_{\mathrm{NP}}H}\rfloor$$

where m_NP_ is nanoparticle magnetic moment and k_B_ is Boltzmann constant. Fitting the equation to the experimental magnetization shows that the M-H curves fitted well with the Langevin model (Figure 3d). Moreover, the M_S_ of IO-MMNs was calculated to be = 3.82 emu/g, which is largely higher than that reported for bulk α-Fe_2_O_3_ (0.3 emu/g) [48]. This can be explained by the presence of maghemite in the sample, which induced an increase of saturation.

Finally, hydrodynamic particle size and zeta potential measurements were recorded by DLS. IO-MMNs dispersed in water indicated an average hydrodynamic radius (D_H_) = 310 ± 12 nm and average zeta potential ξ = −13.6 ± 3.16 mV. The relatively sharp peak at ~300 nm further confirmed the core size obtained by electron microscopy and pinpointed the uniform size distribution of IO-MMNs (PDI ~0.6). All the above results confirm the successful formation of IO-MMN mesostructures and their excellent water dispersity, making them suitable for biological cellular assays.

### 3.2. Drug Loading and Release

**Figure 4:**
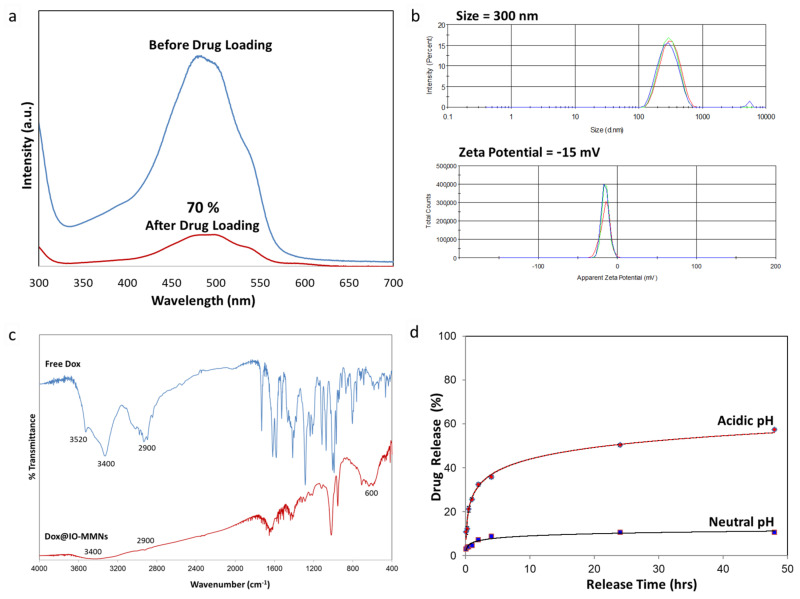
Drug loading and release of Dox@IO-MMNs. (**a**) UV-vis spectroscopy of IO-MMN aliquot before and after Dox loading; (**b**) size and zeta potential distribution graphs showing hydrodynamic sizes (D_H_ = 290 nm) and zeta potential (ξ = −35 mV) of Dox@IO-MMNs dispersed in water; (**c**) FTIR spectra for free Dox (above) and Dox@IO-MMNs (below) showing the peaks associated with Dox and hence its successful loading; (**d**) Dox release profiles of Dox@IO-MMNs at two different pH values, indicating the pH-dependent release of Dox at acidic (4.5) but not at physiological (7.4) pH.

With the IO-MMNs in hand, we first examined drug loading and release of the anticancer chemotherapeutic drug, Dox, typically used in routine chemotherapeutic treatments for patients with various types of cancer (mainly breast, colon, bladder, and leukemia). To this end, loading of Dox into the pores of IO-MMNs was first studied. It was found that up to 70% of Dox (i.e., 140 μg of Dox/mg of MMNs) was loaded onto IO-MMNs, as evident from absorption spectroscopy (Figure 4a).

DLS and zeta potential measurements of Dox@IO-MMNs in aqueous media revealed size (D_H_ ~290 ± 9.9 nm) and zeta potential (ξ = −15 ± 3.25 mV) (Figure 4b), pinpointing that the particle size, uniformity, and surface properties did not significantly change after drug loading. This proves that the drugs are in fact incorporated within the mesopores of IO-MMNs. FTIR spectrum for Dox@IO-MMNs clearly showed the same characteristic absorption peaks for Dox with distinctive bands at ~3400 cm^−1^ due to O-H/N-H and ~2900 cm^−1^ for C-H stretching vibrations (Figure 4c). Importantly, the spectrum showed the appearance of Fe-O stretching at ~600 cm^−1^, confirming the presence of iron oxide, and the disappearance of the band at 1730 cm^−1^, corresponding to carbonyl (C=O) of Dox. This indicates the successful loading of Dox into IO-MMNs via the involvement of -NH_2_, -OH and C=O groups, consistent with our previous reports. Mild shifts in IR spectra upon drug loading along with DLS measurements indicate that no significant structural changes occurred to either the drugs or the IO-MMN constructs upon drug loading.

Next, in vitro drug release studies of Dox@IO-MMNs in PBS buffer was investigated at neutral (7.4) and acidic (4.5) pH values (Figure 4d). While almost negligible amounts of less than 10% drug release was observed at neutral pH, a rather faster but sustained drug release was evident at acidic pH representing ~50–60% of Dox content after 24 to 48 h. From the release studies, it is apparent that Dox is tightly held within the IO-MMN pores due to strong electrostatic interactions and hydrogen bonding between the drugs and particle/pore surfaces at neutral pH. Because of the protonation effect at lower pH values [49], those interactions are broken, causing sustained-release of Dox. This controlled drug-release at neutral pH with faster but sustained release at low acidic pH is particularly appealing for drug delivery systems. It is anticipated that the acidic environment of late endosomes/lysosomes inside the cells will facilitate drug release from IO-MMNs, while the release at normal physiological conditions will be much slower, reducing unwanted side effects [43,49,50]. This relatively slow but controlled release of Dox is desirable to reach cytotoxic concentrations of the drug once internalized inside the cells, achieving efficient and sufficient dosages of the anticancer drug in the process of cancer therapy. Thus, the pH-dependent drug release properties of the IO-MMNs found here make it a promising pharmaceutical carrier for effective therapeutic drug delivery.

### 3.3. Biological Assays

**Figure 5:**
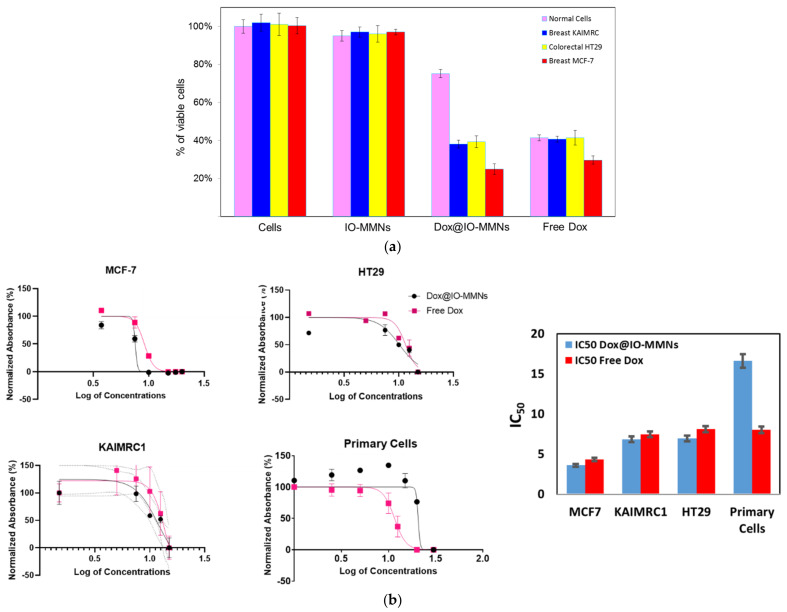
MTT cell viability assays for IO-MMNs, Dox@IO-MMNs, and free Dox with the various types of cancerous and primary normal cells. (**a**) Percentage of viable cells upon incubation with IO-MMNs (35 μg/mL), free Dox (5 μg/mL Dox), or Dox@IO-MMNs (35 μg/mL IO-MMNs, 5 μg/mL Dox) at the same concentrations. (**b**) Dose-dependent non-linear regression curves of the cytotoxicity assays on the different cancer cells as well as on normal primary cells determined using MTT assay. Results show that while free IO-MMNs display no substantial toxicity to any of the tested cells, Dox@IO-MMNs were found to be highly potent to the three cell lines with IC_50_ values ranging from ~3.5 to 7 μg/mL Dox content, and up to 4-fold more toxic as compared to normal cells (IC_50_ ~16.6 μg/mL Dox), being the least sensitive. Free Dox, however, at the same concentrations, equally killed all the tested cells including normal cells. *x*-axis: Log of concentrations expressed in μg/mL; *y*-axis: absorbance at 450 nm. Triplicates were conducted and error bars denote standard deviations.

Cell viability by 3-(4,5-Dimethylthiazol-2-yl)-2,5-diphenyltetrazolium bromide, commonly known as the MTT cytotoxicity assay, was evaluated. While the unloaded free IO-MMNs were not toxic to any of the tested cells up to 75 µg/mL, Dox@IO-MMNs were found to be cytotoxic to both MCF-7 and KAIMRC-1 breast cancer cells (proliferation inhibition IC_50_ = 3.58 µg/mL Dox, and IC_50_ = 6.83 µg/mL Dox respectively) and to HT-29 colon cancer cells (IC_50_ = 6.93 µg/mL Dox) (Figure 5). From the IC_50_ graphs, Dox@IO-MMNs showed ~2-fold enhanced cytotoxicities against the metastatic breast cancer MCF-7 in comparison to KAIMRC-1, probably due to their growth in 3D conformation [51,52]. The potency of Dox@IO-MMNs on MCF-7, KAIMRC-1, and HT-29 was slightly better than the potency of free Dox (IC_50_ = 4.32, 7.44, and 8.09 µg/mL, respectively), which was found potent to the three different cells at almost equivalent inhibitory concentrations (Figure 5).

Nevertheless, primary normal human cells were found to be the least sensitive to Dox@IO-MMNs with IC_50_ = 16.6 µg/mL Dox, while being equally sensitive to free Dox (IC_50_ = 8.01 µg/mL Dox). In general, it is illustrated that Dox@IO-MMNs showed the most cytotoxic effects on the tested cancerous cells with ~2–4-fold enhanced potencies against the breast and colon cancer cells in comparison to normal cells. Importantly, the free Dox at equivalent concentrations killed all the tested cancerous cell lines, including the normal cells, equally. This was observed in the enhanced potencies of Dox@IO-MMNs against the cancerous cells, with the least efficacies towards the normal cells suggest promising capabilities for IO-MMNs to be employed as efficient and selective drug delivery nano-pharmaceutics.

**Figure 6:**
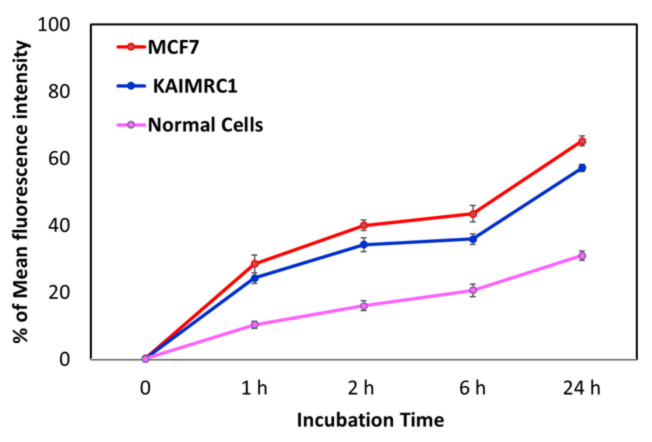
Flow cytometry analysis of FITC-labeled IO-MMNs. Uptake of IO-MMNs by two different cancer cell lines and normal primary cells as measured by fluorescence-activated cell sorting (FACS) after incubating the labeled particles with the cells at different time points. The cellular uptake was slightly higher for MCF-7 compared to KAIMRC-1, with least uptake for normal primary cells. Data are presented as mean values ± standard deviations.

To better understand the observed phenomena, the uptake by the different cell lines was measured using flow cytometry. We labeled IO-MMNs with a fluorescent label “Fluorescein IsoThioCyanate (FITC)” and the uptake of the labeled FITC@IO-MMNs by different cell lines at various time points were then recorded. Upon incubation with the FITC@IO-MMNs, the tested cell types exhibited significant time-dependent cellular uptake, with ~20% observed in the first hour and ~40% after 2 h, but slowly declining thereafter to reach ~60% after 24 h (Figure 6). Interestingly, the FACS results demonstrated an increased uptake of both breast cancer cells ~2-fold greater compared to the primary normal cells with the least uptake observed (61.2% vs. 30.9% after 24 h). This corroborated the obtained MTT cytotoxicity assay results. It is important to note that the fluorescence signals of the incubated cells were measured after thorough washing, indicating that the uptake is not only due to the adherence to the cellular membrane, but rather to the intracellular delivery inside the cells. No significant change in the fluorescent intensity was observed after 24 h, confirming that most of the uptake took place in the first hours, further validating the in vitro release data. This is also consistent with many previous studies, including ours, reporting on the increased uptake of NPs by cancer cells in the first few hours, and gradually slowing down thereafter.

**Figure 7:**
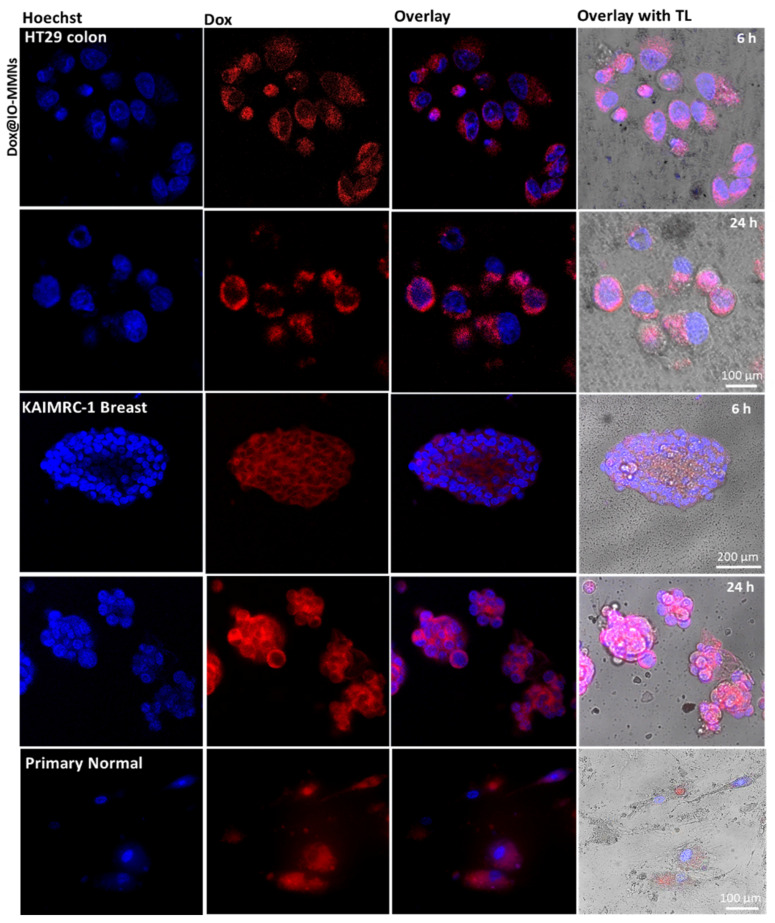
Live confocal microscopy images of HT-29 colon, KAIMRC-1 breast, and primary normal cells incubated with Dox@IO-MMNs (35 μg/mL particles; 5 μg/mL Dox). Left to right: Hoechst nuclei stain (blue), Dox channel (red), overlay of Dox and Hoechst channels, and overlay with transmitted light (TL). The confocal images show the gradual internalization of IO-MMNs inside the cell cytoplasm with time and effective delivery of Dox killing both colon and breast cancer cells but not the primary normal cells. The apoptotic features with irregular membrane blebbings are clearly seen in the cancerous cells, but not in the primary normal cells.

Finally, in order to analyze the intracellular delivery of Dox@IO-MMNs by the different cells, live confocal microscopy imaging without cell fixation was examined. The acquired images confirmed that the cytotoxicity of Dox@IO-MMNs is due to the gradual delivery of Dox inside the cells with time. Following the intracellular IO-MMN uptake, the lower pH inside endosomes/lysosomes facilitates the release of Dox from IO-MMN vehicles, which is then translocated to the nucleus with time causing apoptotic cell death. Confocal images of the three tested cell lines after incubation with Dox@IO-MMNs at same concentrations showed increased red fluorescence of Dox for both colon and breast cancerous cells with time in comparison to normal cells (Figure 7). In cancerous cells, the intense red fluorescence was translocated to the nucleus with typical apoptotic features clearly observed (Figure 7).

**Figure 8:**
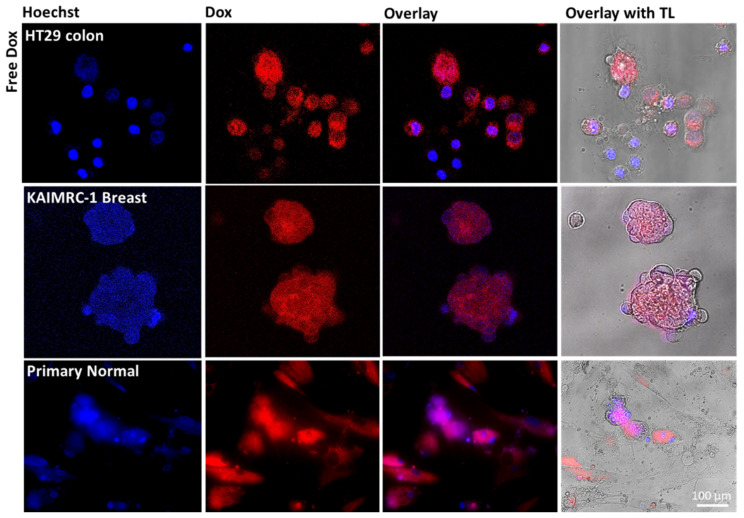
Live confocal microscopy images of HT-29 colon, KAIMRC-1 breast, and primary normal cells incubated with free Dox at equivalent concentration (5 μg/mL Dox). Left to right: Hoechst nuclei stain (blue), Dox channel (red), overlay of Dox and Hoechst channels, and overlay with transmitted light (TL). The confocal images show the direct diffusal of the free drug to the nuclei of all cells, killing both the cancerous and normal cells.

**Figure 9:**
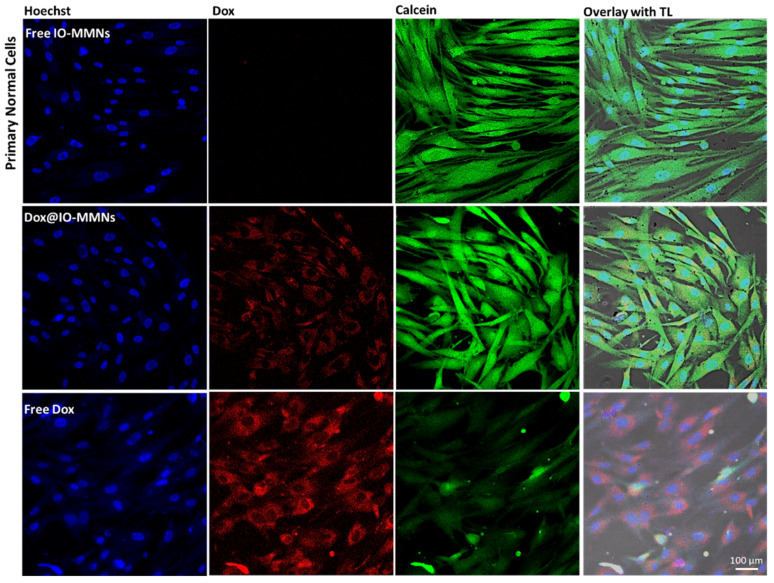
Live confocal microscopy images of primary normal cells after 24 h of incubation with free IO-MMNs, Dox@IO-MMNs, or free Dox (30 μg/mL particles; 4 μg/mL Dox). Left to right: Hoechst (blue) nuclei stain, Dox (red), Calcein (green) viable cells, and overlay of channels with transmitted light (TL). The confocal images noticeably show the viability of primary cells when incubated with both free IO-MMNs and Dox@IO-MMNs, but not with the free drug.

Conversely, for the normal cells, the red Dox fluorescence was found to be mainly located in the cytoplasm, with no noticeable nuclear staining or apoptosis. Notably, when the different cancerous and primary normal cells were incubated for either 6 or 24 h with free Dox at exactly the same concentrations, direct diffusal of free drug to the nuclei of all cells was evident, killing both the cancerous and normal cells concurrently (Figure 8). These observations confirmed the cytotoxicity assay results obtained. Lastly, primary normal cells were treated with Calcein (used to detect viable cells) and incubated with free IO-MMNs, Dox@IO-MMNs, or free Dox at equivalent concentrations. The confocal images remarkably showed the viability of primary cells (depicted by the green fluorescence) when incubated with both free IO-MMNs and Dox@IO-MMNs but not with the free Dox (Figure 9). Collectively, our results strongly suggest the important utilization of IO-MMN as promising vehicles for enhanced and selective anticancer drug delivery. Furthermore, using the IO-MMN vehicle is by itself beneficial, as it immensely decreases the unwanted side effects of free anticancer drugs. This directed payload is promising to enhance the efficacy of drugs in cancer patients with lower doses administrated, thus potentially opening new avenues for in vivo selective image-guided cancer theranostics.

## 4. Conclusions

In conclusion, we synthesized unique iron oxide mesoporous superparamagnetic nanovehicles with relatively high surface areas (*S_BET_* = 70–120 m^2^/g), pore volumes (*V_pore_* = 0.25–0.45 cm^3^/g), and pore size diameters (*d_pore_* = 15–30 nm). Due to their intrinsic and excellent porosity characteristics, IO-MMN can be loaded with anticancer drugs with high loading efficiencies, and then sustainably released inside the cancerous cells in a pH-dependent manner. The drug@IO-MMN formulation described here demonstrated enhanced and selective cytotoxic effects towards different breast and colon cancer cells, with noticeably least potency towards normal primary cells. This IO-MMN platform suggests promising potentials as anticancer nanovehicles for cancer therapeutics and may open new opportunities in effective delivery of drugs, overcoming limitations of conventional cancer therapeutics.

## Data Availability

All data generated in this study are included in this published article. Any other relevant data is available upon request.
